# Apis-Mellifica

**Published:** 1895-05

**Authors:** 


### HOMŒOPATHIC PHYSICIAN,

A MONTHLY JOURNAL OF

HOMŒOPATHIC MATERIA MEDICA AND CLINICAL MEDICINE.

"If our school ever gives up the strict inductive method of Hahnemann, we are lost, and deserve only to be mentioned as a caricature in the history of medicine."—CONSTANTINE HERING.

Vol. XV. MAY, 1895. No. 5.

### EDITORIAL.

APIS-MELLIFICA.—With the present article we give the last of Dr. Lippe's notes concerning Apis.

As has been before said, these editorials will be of no value to the readers of this journal unless they make them their own by memorizing or by noting them in some kind of repertory, so as to have them ready for instant application when called to a case.

In the mouth and throat symptoms of Apis we have the well-known indication, "swelling of the lips, especially the upper lip." This is quite an important characteristic of Apis, said Dr. Lippe in speaking of it; but Calcarea-carbonica and Belladonna also have it. The whole face of the Apis patient swells up, giving the impression that he has been stung by bees.

The pouting upper lip is an indication also for Calcarea. But the Calcarea patient has, at the same time, a swelling of the abdomen like a saucer turned bottom upward, and the whole individual is fat and flabby.

In the Apis patient the lower lip cracks open and the tongue feels scalded. There is burning and stinging of the throat not relieved by drinking water; and there are burning, stinging blisters on the edge of the tongue. Belladonna has burning in the throat with dryness and sense of constriction.

Spongia and Apis both have dryness of the throat without thirst.

This thirstlessness of Apis is one of its prime keynotes. Thirstlessness with dropsy is always a keynote of Apis.

In the provings of Apocynum in *The Guiding Symptoms*, Dr. Hering refers to Apis, and remarks pointedly that Apis "has no thirst in dropsies."

Of the fullness and distention of the abdomen recorded under Apis, Dr. Lippe remarked that it was significant of inflammation of the intestines, and that it was attended with nausea and retching followed by vomiting.

It is similar to Sulphur and Pulsatilla, but the pain of Apis is more intense.

The bloating is also similar to Calcarea-carbonica, Lycopodium, and Silicea. This distention of the abdomen is also significant of dropsy with swelling of ankles extending far up the legs, dyspnœa, diminished secretion of urine, and, of course, *thirstlessness.*

The diarrhœa of Apis is generally painless and is generally in the morning.

In typhus fever, when Apis is indicated, both stool and urine are involuntary.

Apis has tenesmus and discharge of blood after loose stool similarly to Mercurius. The dysentery of Apis is attended with thirstlessness, also.

Apis, Arsenic, Ipecacuanha, and Veratrum all have diarrhœa and vomiting. Apis has continual oozing from the anus of dark, bloody fluid. This symptom recalls the peculiar symptom of Phosphorus: "Discharge of mucus out of the wide-open anus." This is Dr. Lippe's keynote for Phosphorus.

Apis has strangury like Cantharis. This occurs especially in children. There is burning and smarting along the urethra as if it were scalded. The patient is troubled with continuous desire to urinate.

A striking characteristic of Apis is that *the last drops of urine burn and smart when passed.*

Apis is a prominent remedy for dropsy of genitals occurring after scarlet fever.

Apis has pressing-down pain in the uterus. This symptom is similar to Natrum-carbonicum and Nux-vomica.

Apis has also dysmenorrhœa in young girls. This is attended with congestion to the head.

The prime keynote of Apis is its burning, stinging pains. Nitric acid has stinging pains, but Apis has *burning*, stinging pains, and so has Gelsemium.

Apis generally has sensitiveness to pressure, but the headache of Apis is relieved by pressure.

The Apis patient sleeps late in the morning like Nux-vomica; but he is harder to arouse. His sleep is a species of stupor.

The chill of Apis is worse from external heat and the fever is attended with burning of the hands and feet. The perspiration of Apis smells like musk.

The Apis patient sleeps after the fever paroxysm, and the Nux-vomica patient sleeps before it—that is, between the chill and fever.

Cactus-grandifloris and Lachesis have sleep during the fever stage of the paroxysm.

The Apis patient is worse from the heat of a room. This is similar to Pulsatilla. The Apis patient must have his window open. This is similar to Pulsatilla and Sulphur.

All these notes, the reader will bear in mind, are simply Dr. Lippe's comments. At the risk of much repetition we add Dr. Guernsey's keynotes.

The parts have a stinging like bee-stings. Eruptions on genitals stinging like bee-stings. Profuse uterine hemorrhage and heaviness in the abdomen. Faintness. Great uneasiness and yawning. Red spots like bee-stings on the skin. Menorrhagia with heaviness of abdomen. Enlargement of right ovary with pain in left pectoral region and cough. Mutual sympathy between lungs and ovaries.

Absence of thirst in dropsy.

Scanty urine and absence of thirst. Obstinate constipation. Stools seldom and difficult, with stinging pains and sensation in abdomen of something tight which would break if too much effort were used. Frequent waking at night with violent screams, especially of infants during dentition. Red spots over infant's body, here and there. The child is restless and screams out in its sleep. Frequent bloody stools without pain.

Waxy paleness of feet and legs, which are much swollen. The child screams out suddenly as if from stinging pains. Erysipelatous eruption, inclining to spread all over the child and to become gangrenous. Red, inflamed, raised patches of urticaria with burning, stinging pains.

